# Pigs receiving daily tailored diets using precision-feeding techniques have different threonine requirements than pigs fed in conventional phase-feeding systems

**DOI:** 10.1186/s40104-019-0328-7

**Published:** 2019-02-22

**Authors:** Aline Remus, Luciano Hauschild, Etienne Corrent, Marie-Pierre Létourneau-Montminy, Candido Pomar

**Affiliations:** 1 Sherbrooke Research and Development Centre, Agriculture and Agri-Food Canada, Sherbrooke, Quebec, J1M 0C8 Canada; 2 Département des sciences animales, Université Laval, Quebec City, Quebec, G1V 0A6 Canada; 3 School of Agricultural and Veterinary Studies (FCAV), Department of Animal Science, University of São Paulo State (UNESP), Jaboticabal, São Paulo, 14883-108 Brazil; 4 Ajinomoto Animal Nutrition Europe, Paris Cedex 17, F-75817 France

**Keywords:** Amino acid body composition, Dose-response, Ideal protein profile, Lysine, Threonine

## Abstract

**Background:**

There is large variation in amino acids requirements among pigs, hence feeding pigs individually with daily tailored diets or in groups with a single feed may require different levels of nutrients. Thus, the response to different threonine levels (70%, 85%, 100%, 115%, and 130% of the ideal threonine:lysine protein ratio of 0.65) was studied in growing pigs raised in a conventional group phase-feeding (GPF) system or fed individually using individual precision-feeding (IPF) techniques. In a 21-day trial, 110 barrows (25 ± 0.80 kg body weight) were housed in the same room and fed using electronic feeders. Five pigs per treatment were slaughtered at the end of the trial.

**Results:**

Threonine intake increased linearly for the IPF and GPF pigs (*P* < 0.05). Lysine intake was similar across the treatments. Average daily gain, gain:feed ratio, and protein deposition were affected linearly by threonine level (*P* < 0.05) in both feeding systems. Protein deposition in the GPF pigs was maximized at 150 g/d and a 0.65 threonine:lysine ratio, whereas protein deposition increased linearly in the IPF pigs. Plasma Met and serine levels were 11 and 7% higher, respectively, in the IPF pigs than in the GPF pigs (*P* < 0.05). Dietary threonine increased (*P* < 0.05) threonine concentration in the longissimus dorsi in a quadratic manner in the IPF pigs, whereas there was no effect in the GPF pigs. Longissimus dorsi collagen decreased as dietary threonine increased in the IPF and GPF pigs (*P* < 0.10). Carcass muscle crude protein was 2% higher in the GPF pigs than in the IPF pigs (*P* < 0.05).

**Conclusions:**

Individual pigs are able to modulate growth and the composition of growth according to threonine intake. The average amino acid ratio value that is currently used for GPF cannot be used for IPF.

## Background

Pigs are usually fed in groups with the same diet provided during each feeding phase, and the composition of the diet is adjusted to the estimated nutrient requirements of a representative animal in the group. These requirements are often estimated using factorial methods in which the average pig is taken as the reference for the population (e.g., National Research Council, 2012 [1]). However, pigs have different requirements, and these requirements change over time [2]. Optimal responses in conventional group phase-feeding (GPF) systems are, however, obtained with levels of nutrients that satisfy the requirements of the most demanding animals in the group, because for most nutrients, underfed pigs exhibit reduced growth performance, whereas overfed ones exhibit near optimal performance [2, 3]. Indeed, most of the pigs receive more nutrients than they need to express their growth potential [2]. Feeding pigs with daily tailored diets using individual precision-feeding techniques (IPF) is proposed to alleviate the limitations of group-feeding systems [4, 5]. Individual lysine (Lys) requirements are estimated in IPF systems according to each pig’s daily feed intake, body weight (BW), and daily gain patterns [2]. Other amino acid (AA) requirements are established according to a recognized ideal AA profile using Lys as the reference AA. It has been demonstrated that, in relation to conventional GPF systems, precision feeding can reduce Lys intake by 26%, nitrogen excretion by 30%, and feeding costs by 10% [6, 7]. The ability of the proposed method to estimate individual pig Lys requirement has been validated [8, 9], but no validation of the method’s estimation of other AA requirements, which today are estimated using a conventional ideal AA profile, has been performed. It has been recently observed, however, that pigs fed daily tailored diets might have higher methionine (Met):Lys ratios than pigs in GPF systems do [10].

Threonine (Thr) is often the second-limiting AA in conventional commercial diets, and feeding pigs AA deficient diets limit protein deposition (PD) and affects tissue protein composition [11, 12]. Thus, Thr deficiency might lead to the synthesis of proteins with less Thr and a reduction of the Thr concentration in the overall body muscles [13]. Because IPF significantly reduces Lys intake, we hypothesized that the ideal AA profile may differ between IPF and GPF systems and that using the current AA recommendation may limit PD and change plasma and muscle AA concentrations in precision-fed pigs. The aim of this study was to evaluate metabolic changes due to feeding pigs with increasing levels of dietary Thr (70%, 85%, 100%, 115%, or 130% of the estimated ideal standardized ileal digestible [SID] Thr:Lys ratio of 0.65 [14]) on animal growth performance and on plasma and body protein AA concentrations in IPF and GPF systems.

## Methods

### Animals, housing, and management

Animals were cared for in accordance with a recommended code of practice [15] and the guidelines of the Canadian Council on Animal Care [16], and the animal trial was approved (Case No. 478) by the Ethical and Animal Welfare Committee of Agriculture and Agri-Food Canada’s Sherbrooke Research and Development Centre (Sherbrooke, QC, Canada).

A total of 110 healthy barrow pigs of the same high-performance genotype (Fertilis 25 × G-Performer 8.0; Geneticporc Inc., St-Gilbert, QC, Canada) were shipped to the swine complex at the Sherbrooke Research and Development Centre. The pigs were allocated to one of two 76-m^2^ pens with concrete slat floors in the same mechanically ventilated room. The pigs each had an electronic chip placed in their ear to give them access to the feeders. Between their arrival and the start of the trial, the pigs were fed commercial growing diets. Water was provided with low-pressure nipple drinkers, and feed was provided individually ad libitum throughout the adaptation period (14 d) and experimental period (21 d) with 10 feeding stations (Automatic and Intelligent Precision Feeder; University of Lleida, Lleida, Spain). The temperature of the room was decreased gradually from 22 °C when the piglets arrived to 18 °C at the end of the experimental period to ensure thermoneutral conditions. The photoperiod consisted of 12 h of light and 12 h of darkness. The pigs’ health status was checked daily. This check included daily observations of DFI records and monitoring for the presence of diarrhea and for other signs of health disorders. Body temperature was measured when distress conditions were observed, and pigs were treated in accordance with veterinarian recommendations when necessary.

The pigs (25 ± 0.80 kg BW) were assigned randomly to the treatments in two complete blocks according to a 2 × 5 factorial arrangement, with the main factors being (1) two feeding systems (IPF or GPF), and (2) five Thr levels (70%, 85%, 100%, 115%, or 130% of the estimated ideal Thr:Lys ratio of 0.65 [14]). The experimental unit was the individual pig, and each treatment included 11 replicates. Each of the two complete blocks included 55 pigs, and the blocks started the experimental period one week apart. Pigs within each block were housed in the same pen. Individual transponder codes allowed the feeders to identify individual pigs, record feed intake data and the feeds to be provided to each pig according to the assigned feeding system and Thr level. In each single-space feeder, precision Archimedes screw conveyors delivered and simultaneously blended volumetric amounts of up to four feeds stored in independent containers located in the top of the feeder [17]. The feeder identified each pig when the feed demand was made, and the feeder read the specific treatment formula for that pig, mixed the feed in accordance with the assigned treatment, and dropped the feeds into the feeder tray. A time lag between services was set in accordance with the pig’s BW and feed intake. All the feeders were designed to provide meals to all the animals, regardless of the treatment. Because of this feature, all the animals could be housed in the same pen [6, 18] and each animal could be considered an experimental unit.

### Feeding programs, nutritional requirements, and diets

Data from high-performance pigs from previous trials completed at the Sherbrooke Research and Development Centre were used as the reference population for calculating the pigs’ Lys requirement to formulate the feeds (named A1, A2, B1, and B2) (Table 1). The formulation of these feeds was performed using each ingredient’s SID AA content obtained by determining the product of its tabulated total AA content [1] and the SID value in the INRA-AFZ tables [19]. The four experimental feeds were formulated to contain similar net energy concentrations and AA profiles for AA other than Thr. The AA were provided 10% above the ideal AA:Lys ratios: 30% for Met [13], 60% for Met + cysteine [13], 65% for Thr [14], 22% for tryptophan [20], 70% for valine (Val) [21], 51% for isoleucine (Iso) [22], 100% for leucine (Leu) and 32% for histidine (His) [22] and 42% for arginine (Arg) [1], whereas Lys was provided 10% under the estimated requirements [2]. Feeds A1 and A2 were formulated to satisfy the requirements for minerals and AAs other than Thr of the most demanding pigs in the reference population, and feeds B1 and B2 were formulated to satisfy the requirements for minerals and AAs other than Thr of the less demanding pigs in the reference population [2, 6, 7]. However, feeds A1 and B1 were formulated to provide 130% of the optimal Thr:Lys level, and feeds A2 and B2 were formulated to provide 70% of the optimal Thr:Lys level. Dietary phosphorus and calcium requirements were estimated according to the National Research Council [1]. Microbial phytase was not added, but the calcium:digestible phosphorus ratio was kept constant.

**Table 1:** Ingredient and chemical composition (as-fed basis) of the experimental feeds (A1, A2, B1, and B2)

Item	A1	A2	B1	B2
*Ingredients g/kg*				
Corn	533	538	537	538
Soybean meal (48%)	173	173	–	–
Wheat	150	150	100	100
Canola meal	47	47	–	–
Corn gluten meal **+** linseed meal^a^	33	33	–	–
Corn starch	–	–	156.3	156.3
Fat	16	16	35	35
Oat hulls	–	–	143	143
Limestone	12	12	8	8
Monocalcium phosphate	10	10	8	8
Salt	5.50	5.50	4.80	4.80
Anti-mould	1.00	1.00	1.00	1.00
Choline chloride (75%)	0.20	0.20	0.20	0.20
Lysine sulfate (70%)	6.70	6.70	2.80	2.80
l-threonine	4.50	–	1.20	–
dl-methionine	2.30	2.30	0.20	0.20
l-valine (96.5%)	2.10	2.10	0.20	0.20
Vitamin mineral premix^b^	2.00	2.00	2.00	2.00
l-tryptophan	1.10	1.10	0.30	0.30
l-isoleucine	0.70	0.70	0.20	0.20
*Chemical composition, %*				
Dry matter	90.85	91.25	92.99	92.67
Crude fat	6.79	6.74	7.88	8.44
Crude protein	19.85	19.88	7.5	6.88
Acid detergent fibre	3.87	4.02	6.32	6.51
Neutral detergent fibre	8.80	8.63	13.58	14.12
Total calcium	0.72	0.72	0.50	0.49
Total phosphorus	0.64	0.64	0.40	0.40
Digestible phosphorus^c^	0.35	0.35	0.27	0.27
SID^d^ isoleucine	0.67	0.69	0.22	0.21
SID leucine	1.34	1.39	0.64	0.59
SID lysine	1.07	1.07	0.34	0.33
SID methionine	0.53	0.53	0.16	0.14
SID methionine + cysteine	0.72	0.72	0.24	0.20
SID phenylalanine	0.75	0.77	0.28	0.26
SID serine	0.80	0.80	0.30	0.26
SID threonine	0.98	0.58	0.31	0.19
SID valine	0.89	0.89	0.29	0.27
Calculated net energy, MJ/kg	13.43	13.49	13.63	13.65

^a^Mix of corn gluten meal and linseed meal (Shur-Gain Canada)

^b^Supplied per kilogram of diet (as-fed basis): vitamin A, 11,400 IU; vitamin D, 1140 IU; vitamin E, 35 IU; vitamin K, 2 mg; vitamin B_12_, 30 μg; niacin, 20 mg; pantothenic acid, 15 mg; pyridoxine, 2 mg; thiamine, 2 mg; copper, 122 mg; iodine, 0.3 mg; iron, 100 mg; manganese, 63 mg; selenium, 0.3 mg; and zinc, 152 mg

^c^Digestible phosphorus, standardized ileal digestible amino acids, and metabolizable energy were estimated from the analyzed total amino acid and crude energy content in feed and from values in the INRA-AFZ tables [19]

^d^SID, standardized ileal digestible

Dietary treatments for the IPF and GPF pigs were obtained by blending the four experimental feeds in the required proportions. For the IPF pigs, the required daily concentration of SID Lys was estimated with a mathematical model using individual feed intake and weekly BW information [2]. With this historical information, the empirical component of the model estimated, for each pig, the expected BW, DFI, and weight gain for the starting day on which the pig would receive the calculated feed blend. Thereafter, the mechanistic component of the model used these three estimated variables to calculate, by means of a factorial method, the optimal concentration of Lys that should be offered that day to each pig in the herd to meet its requirements. This method of estimating nutrient requirements was described previously [2, 6] and validated in three earlier studies [7–9]. The use of this model allowed each pig in the IPF system to receive, each day, a diet tailored to its Lys requirement. In the GPF system, Lys requirement was estimated by assuming that the population requirements were those of the 80^th^-percentile pig in the group at the beginning (average of 3 d) of the phase [10, 23] and maintained constant for all pigs through out the feeding phase. However, SID Lys supplies were decreased by 10% to ensure that Lys was the second-limiting AA [24], whereas the other AAs except Thr were provided 10% above the estimated levels. Threonine was provided at the assigned treatment level. The AA ratios were calculated in the same way in both feeding systems and kept constant throughout the experiment.

### Experimental measurements

#### Performance

The pigs were weighed at arrival and three times during the adaptation period to calibrate the model before the experimental protocol was applied. Animal performance was evaluated through average daily feed intake (ADFI) (kg/d), average daily gain (ADG) (kg/d), gain:feed ratio (G:F) (kg/kg), SID Lys intake (g/d), SID Thr intake (g/d), total body PD (g/d), PD in daily gain (%), and total body lipid deposition (LipD) (g/d). Total body fat and lean content were measured by dual X-ray absorptiometry (DXA) on d 1 and 21 of the trial with a densitometer device (GE Lunar Prodigy Advance, Madison, WI, USA). The pigs were scanned in the prone position using the total-body scanning mode of the manufacturer-provided software (Lunar enCORE Software, version 8.10.027). Anesthesia was induced with sevoflurane (7%) and maintained with isoflurane (5%) during the scans.

#### Blood sampling

Blood samples were taken on d 21 after 10 h of fasting. Samples from the jugular vein were collected in Vacutainer tubes with EDTA anticoagulant for enzymatic and biochemical analyses or with sodium heparin for the AA analysis. The time between sampling and centrifugation did not exceed 1 h, during which the samples were kept on ice. The blood samples were centrifuged for 15 min at 1000×*g* at 4 °C. For AA analysis, 20 μL of standard enriched AAs was added to the samples within 30 min after centrifugation. All plasma samples were kept at − 20 °C during the sampling day and stored at − 80 °C at the end of the day.

#### Organ and muscle sampling

Five pigs per treatment were randomly chosen and slaughtered in a commercial slaughterhouse between d 22 and 28, and the treatments were maintained during this period. Each pig carcass was scalded and scraped, and the eviscerated carcass was split longitudinally, with the head and feet kept on it. The right side of the carcass was dissected, and the head and feet were discarded. The longissimus muscle was separated from the loin cut. The liver and the small intestine (washed and free of mesentery) were collected. All samples were sealed in separate vacuum plastic bags and stored for a maximum of 2 months at − 20 °C until sampling. The liver and small intestinal tissue were ground twice and sampled. The pool of dissected muscles was cut into cubes and mixed for grinding. The longissimus dorsi and a pool of all the other muscles were ground four times and sampled. All the samples were freeze-dried and stored at − 80 °C until analysis.

#### Chemical and biochemical analyses

Two replicates of each sample were analyzed using the Association of Official Analytical Chemists [25] standard methods for lyophilization (method 938.18), determination of protein in the feed, liver, and small intestinal tissue (method 992.15) (Kjeltec 2400; FOSS Tecator, Hillerød, Denmark), and determination of lipids (method 991.36) (Soxtec 2050 Automated Extraction System; FOSS, Höganäs, Sweden). Crude protein (CP), collagen, and fat in the longissimus dorsi and in the pool of carcass muscles were determined by near-infrared transmittance (method 2007.04) (FOSS FoodScan near-infrared spectrophotometer), and dry matter (DM) (method 950.46) and ash (method 920.153) were also determined. Concentrations of AAs in plasma were determined as suggested by Calder et al. [26]. Thus, the pool of carcass muscles and of longissimus dorsi muscle were first lyophilized, and the samples were hydrolyzed with a solution of HCl 6 mol/L and 0.1% phenol in a block digester at 110 °C for 24 h. A mixture of standard isotopes (200 μL) was added to the samples. A solution of 100 μl of DL-dithiothreitol (15.4 mg/mL of water) was added to the sample which for 30 min at room temperature. Afterwards, the samples were passed through columns (Poly-Prep 731–1550; Bio-Rad, Brossard, QC, Canada) prepared with 0.8 cm (0.4 mL) of resin (Dowex 50WX8–200 ion exchange resin; Sigma-Aldrich, Oakville, ON, Canada). The columns were rinsed twice with 2 mL of ultra-pure water. Amino acids were recovered by adding 2 mL of NH_4_OH_2_N to the columns. The columns were rinsed with 1 mL of ultra-pure water and left to drain into vials. The vials were covered with Parafilm and vortexed. The samples were frozen at − 80 °C and lyophilized. The vials were rinsed with 250 μL of ultra-pure water, and the contents were transferred to a reaction vial (Pierce 13,221;). The contents of the reaction vials were dried with nitrogen at 90 °C for about 20 min, and 20 μL of DL-dithiothreitol (15.4 mg/mL) and 80 μL of NH_4_OH_2_N were added to the samples. The samples were left to stand for 30 min at room temperature and were then dried with nitrogen at 90 °C for 20 min before being derived with 60 μL of MTBSTFA:DMF 1:1 (MTBSTFA: Aldrich 394,882, DMF: Aldrich 27.054–7; Oakville, ON, Canada).). The samples were heated at 90 °C for 35 min and transferred to vials for gas chromatography (Agilent 5182–0714 vials; Agilent Technologies, Saint-Laurent, QC, Canada). All AA samples were measured by gas chromatography–mass spectrometry (Agilent Technologies 7890B gas chromatograph system coupled to an Agilent Technologies 5977A mass selective detector). The immunoglobulin G (IgG) content was determined by means of enzyme-linked immunosorbent assay (ELISA) kits (Pig IgG ELISA Quantitation Set, ref. E100–104; Bethyl Laboratories, Inc., Montgomery, TX, USA). The biochemical and enzymatic analyses of plasma were performed with an automatic analyzer (Beckman DxC 600; Beckman Coulter, Mississauga, ON, Canada) by a dedicated external laboratory (Faculté de médecine vétérinaire, Université de Montréal, Saint-Hyacinthe, QC, Canada).

#### Calculations and statistical analysis

Total pig weight gain was calculated as the difference between the weight measured at the beginning of the trial and the weight measured at the end of the trial. The SID Lys, SID Thr, and CP intakes were obtained for each pig by tallying the daily amount of nutrients provided by each of the blended feeds that were served. Lysine retention and Thr retention were estimated by assuming that 6.9% of body protein is Lys [27] and 3.7% of body protein is Thr [28]. The availability of these AAs for protein synthesis was estimated by removing from the SID pool the amounts used for maintenance. Lysine and Thr maintenance requirements were estimated by adding together the basal endogenous losses, the losses related to desquamation in the digestive tract, and the losses related to the basal renewal of body proteins [29]. Lysine efficiency of utilization and Thr efficiency of utilization were calculated by dividing the corresponding retained amount by the available AA intake. The DXA body lean and fat masses were converted to their protein and lipid chemical equivalents [30]. Protein deposition in gain (%) was calculated by dividing the PD by the ADG. Nitrogen excretion values were obtained by subtracting the respective nutrient retention and intake values.

Performance and carcass data were analyzed as a 2 × 5 factorial arrangement using a mixed model in SAS (version 9.4; SAS Institute Inc., Cary, NC, USA). The main effects were the feeding system, the Thr level, and their interaction, and the block was considered a random effect. The assumption of normal distribution of variables was checked using the Cramer–von Mises test within the UNIVARIATE procedure of SAS. The uncertainty in the estimate of the means of the data was expressed as the maximum standard error (MSE), and a *P*-value less than 0.05 was considered to be statistically significant, whereas a *P*-value less than 0.10 was considered a tendency. Differences between individual treatments were compared with polynomial contrasts. The optimal Thr:Lys ratio was estimated for each feeding program using the NLIN procedure of SAS.

## Results

All but six of the pigs consumed feed and gained weight in accordance with the expected performance of the genetic line. Three of those six pigs had low feed intake, low ADG, and recurrent fever during the adaptation period. Three other pigs were removed from the trial, one because of a severe inflammatory foot problem and two because of respiratory problems unrelated to the trial. All those pigs were treated for their specific problem and isolated, and their data were not considered in the analysis. Thus, the performance data presented in this paper come from 10 pigs for the IPF treatments with 70%, 115%, and 130% of the ideal Thr:Lys ratio (0.65) and the GPF treatment with 85% of that ratio, 8 pigs for the IPF treatment with 85% of that ratio, and 11 pigs for all the other treatments.

### Growth performance, nutrient intake, and nitrogen balance

During the trial, ADFI, SID Lys intake, CP intake, PD in gain, LipD, final BW, and nitrogen excretion were not affected by Thr levels or feeding system (Table 2). Average daily gain, G:F, SID Thr intake, Lys efficiency of utilization, PD, and nitrogen retention increased linearly (*P* < 0.05) and Thr efficiency of utilization decreased linearly (*P* < 0.05) with the level of dietary Thr. However, growth performance, nutrient intake and N balance were not affected by feeding system. No interactions between Thr level and feeding system were observed.

**Table 2:** Initial and final animal body composition, growth performance, and nutrient efficiency of growing barrows (25 to 42 kg body weight) fed different levels of threonine (70%, 85%, 100%, 115%, and 130% of the ideal threonine:lysine ratio of 0.65) in an individual precision-feeding (IPF) system or a group phase-feeding (GPF) system

Parameter	IPF					GPF						*P*-value^2^		
	70	85	100	115	130	70	85	100	115	130	MSE^1^	Thr	FS	Thr× FS
Number of observations	10	8	11	10	10	11	10	11	11	11				
*Initial conditions*														
Body weight, kg	26.0	26.2	25.6	25.2	26.0	26.7	25.7	25.8	25.7	26.2	0.8	0.40	0.49	0.84
Body protein, kg	3.94	3.96	3.83	3.76	3.93	4.06	4.00	3.91	3.87	3.97	0.17	0.23	0.18	0.99
Body lipids, kg	1.18	1.19	1.16	1.14	1.17	1.21	1.20	1.17	1.16	1.19	0.03	0.16	0.23	1.00
*Final conditions, growth performance, and nutrient efficiency*														
Body weight, kg	39.54	40.45	41.47	41.59	43.45	40.80	42.48	42.06	41.74	42.28	1.09	0.11	0.37	0.57
Body protein, kg	6.59	6.68	6.83	6.94	7.28	6.86	6.95	7.04	6.98	7.12	0.23	0.16	0.31	0.76
Body lipids, kg	2.76	2.75	2.71	2.56	2.61	2.76	2.89	2.73	2.61	2.59	0.23	0.64	0.72	0.99
Average daily feed intake, kg/d	1.44	1.46	1.46	1.63	1.50	1.51	1.40	1.49	1.48	1.41	0.14	0.41	0.35	0.47
Average daily gain, kg/d	0.64	0.67	0.76	0.80	0.83	0.68	0.73	0.78	0.77	0.76	0.04	0.01^†^	0.63	0.17
G:F,^3^kg/kg	0.46	0.47	0.51	0.51	0.56	0.45	0.49	0.52	0.52	0.56	0.04	< 0.001^†^	0.64	0.87
SID^4^lysine intake, g/d	11.5	12.3	12.2	13.3	12.9	13.0	12.0	12.8	12.7	12.1	1.3	0.63	0.86	0.22
SID threonine intake, g/d	6.3	7.9	8.9	11.0	11.5	7.1	7.6	9.3	10.2	11.4	0.9	< 0.001^†^	0.99	0.33
Threonine efficiency,^5^%	84	68	65	56	54	75	68	65	57	55	0.07	< 0.001^†^	0.53	0.46
Lysine efficiency,^6^%	80	78	87	85	93	73	78	88	88	94	0.09	< 0.001^†^	0.83	0.77
Protein deposition, g/d	126.2	129.7	141.4	151.1	159.5	130.9	143.1	149.7	148.5	150.2	8.3	< 0.001^†^	0.54	0.59
Protein in gain, %	19.0	19.1	19.1	19.2	19.3	19.0	19.2	19.4	19.4	19.6	0.3	0.43	0.25	0.99
Lipid deposition, g/d	74.8	74.1	74.7	68.3	68.4	74.2	81.0	74.4	69.0	66.8	10.1	0.70	0.84	0.99
*Nitrogen balance*														
Crude protein intake, g/d	222.3	238.4	236.2	258.2	248.6	250.2	230.1	247.0	244.6	234.0	19.48	0.56	0.95	0.22
Efficiency of nitrogen retention, %	55.34	54.68	60.53	59.07	64.51	51.25	54.66	61.25	61.08	65.25	4.77	< 0.001^†^	0.94	0.80
Nitrogen excretion, g/d	16.34	17.39	14.90	16.17	14.26	18.55	16.60	15.58	15.40	13.41	2.96	0.05^‡^	0.91	0.70

^1^MSE, maximum standard error

^2^Thr, level of threonine; FS, feeding system; L × Thr, interaction between level of threonine and feeding system; ^†^linear effect for Thr; ^‡^tendency for a linear effect for Thr

^3^G:F, gain:feed ratio

^4^SID, standardized ileal digestible

^5^Threonine (Thr) efficiency = {(PD × 0.037) − [0.313 g Thr/kg dry matter × DFI + (0.0033 g Thr/kg^0.75^ d × BW^0.75^) + (0.0138 g Thr/kg^0.75^ d × BW^0.75^)]}/SID Thr intake, where PD is protein deposition, DFI is daily feed intake, and BW is body weight

^6^Lysine (Lys) efficiency = {(PD × 0.069) − [0.330 g Lys/kg dry matter × DFI + (0.0045 g Lys/kg^0.75^ d × BW^0.75^) + (0.0239 g Lys/kg^0.75^ d × BW^0.75^)]}/SID Lys intake, where PD is protein deposition, DFI is daily feed intake, and BW is body weight

### Estimation of optimal Thr:Lys ratio

**Fig. 1:**
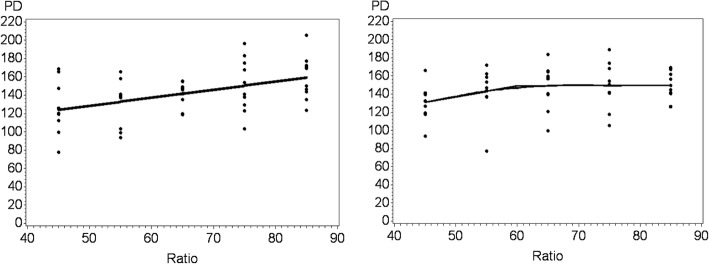
Protein deposition (PD) (g/d) as a function of standardized ileal digestible threonine:lysine ratio according to the linear-plateau and quadratic-plateau models for pigs (25–42 kg BW) in an individual precision-feeding system (right plot) or a group phase-feeding systems (left plot)

Protein deposition, ADG, and G:F were the criterion responses used to estimate the optimal levels of dietary Thr in pigs fed in the IPF and GPF systems (Table 3). These variable responses were preferred because they are directly affected by the AA supply. Increasing the Thr:Lys ratio in the IPF pigs increased the response variables under study, which prevented identification of the optimal ratio. For the pigs raised in the GPF system, however, the breakpoint of the linear-plateau model was observed at Thr:Lys ratios of 60.2%, 64.9%, and 68.6% for PD, ADG, and G:F, respectively, whereas the breakpoint of the quadratic-plateau model was observed at Thr:Lys ratios of 68.2%, 71.1%, and 70.6% (Fig. 1). Thus, in relation to the optimal Thr:Lys ratios obtained with the linear-plateau models for maximum PD, the ideal ratio increased by 8% when ADG was optimized and by 15% when G:F was optimized. These increases on requirements were of 4% when the quadratic-plateau were compared to linear-plateau model in both maximal ADG and G:F. A large variation was found within treatment, and in IPF only 24% (*R*^2^ = 0.24) and in GPF only 20% (*R*^2^ = 0.20) of the variability in the data is explained by the AA ratio.

**Table 3:** Non-linear model parameters between the independent response variables (protein deposition, average daily gain, and gain:feed ratio) and the threonine:lysine ratio in an individual precision-feeding (IPF) system and a group phase-feeding (GPF) system estimated with a linear-plateau model and a quadratic-plateau model

Feeding system	Response^b^	Model parameter^a^							
		U	SEe	R	SEe	L	SEe	*P*-value	RSE
*Linear-plateau model*									
IPF	PD	−0.873	0.25	85.4	6.91	159.5	–	0.00	24.33
	ADG	0.00505	0.002	82.2	11.37	0.8295	0.04	0.00	0.12
	G:F	–	–	–	–	–	–	–	–
GPF	PD	−1.2239	0.99	60.2	9.89	149.5	3.76	0.07	21.61
	ADG	−0.00376	0.001	64.9	24.01	0.77	0.02	0.24	0.12
	G:F	−0.0056	0.003	68.6	6.45	0.5362	0.01	0.03	0.08
*Quadratic-plaateau model* ^*c*^									
GPF	PD	−0.0347	0.059	68.2	19.82	149.5	4.28	0.07	21.61
	ADG	−0.00011	0.0003	71.1	28.51	0.7698	0.03	0.25	0.12
	G:F	−0.00012	0.0002	70.6	17.33	0.5387	0.02	0.03	0.08

^a^U, fit intercept; SEe, standard error of the estimation; R, parameter corresponding to the standardized ileal digestible threonine:lysine ratio required to reach the plateau; L, average response estimated by the model; RSE, residual standard error

^b^PD, protein deposition (g/d); ADG, average daily gain (kg/d); G:F, gain:feed ratio (kg/kg); ^c^Model not converged for IPF

### Biochemical and enzymatic responses in plasma

Plasma creatinine (μmol/L), IgG (μg/mL), and creatine kinase (CK) (U/L) were not affected by feeding system or Thr level (*P* > 0.10) (Table 4). Plasma albumin (g/L) increased (*P* < 0.05) linearly within IPF and it was not affected in the GPF pigs. Plasma total protein (g/L) increased linearly with the increase in Thr levels (*P* < 0.05) but were not affected by feeding system. C-reactive protein (CRP) (μg/mL) increased (*P* < 0.05) in a linear manner in the IPF pigs and in a quadratic manner in the GPF pigs. Alanine aminotransferase (ALT) (U/L) increased (*P* < 0.05) linearly in the IPF pigs and showed a cubic increase in the GPF pigs. Aspartate aminotransferase (AST) (U/L) tended (*P* < 0.10) to increase linearly as dietary Thr increased and tended (*P* < 0.10) to be 8% higher in the IPF pigs than in the GPF pigs. Lactic acid dehydrogenase (LDH) (U/L) tended to be 9% higher in the IPF pigs than in the GPF pigs. Urea (μmol/L) decreased (*P* < 0.05) in a quadratic manner in both feeding systems.

**Table 4:** Plasma free amino acid concentrations of growing barrows pigs (25 to 42 kg body weight) fed different levels of threonine (70%, 85%, 100%, 115%, and 130% of the ideal threonine:lysine ratio of 0.65) in an individual precision-feeding (IPF) system or a group phase-feeding (GPF) system

Parameters	IPF					GPF						*P*-value^b^		
	70	85	100	115	130	70	85	100	115	130	MSE^a^	Thr	FS	Thr × FS
Number of observations	10	8	11	10	10	11	10	11	11	11				
*Essential amino acids, μmol/L*														
Arginine	215.26	210.37	222.60	212.17	208.10	216.69	226.5	217.58	195.36	210.64	18.40	0.87	0.98	0.92
Histidine	54.23	41.64	39.29	44.31	30.26	58.55	45.08	33.79	35.65	35.50	4.05	< 0.001^e^	0.92	0.18
Isoleucine	89.37	78.62	93.25	82.33	88.10	84.62	85.85	82.43	83.39	83.93	5.35	0.67	0.43	0.37
Leucine	148.46	159.01	169.55	166.34	155.39	154.52	153.0	153.95	156.69	157.46	7.62	0.27	0.18	0.25
Lysine	136.93	80.90	75.53	59.26	76.68	125.19	70.60	64.79	62.92	64.32	11.86	< 0.001^d^	0.17	0.89
Methionine	58.56	51.48	47.48	48.34	51.24	46.68	46.42	44.62	51.22	40.38	4.71	0.44	0.04	0.37
Phenylalanine	64.69	70.51	61.73	58.04	61.18	58.25	59.41	59.03	63.69	62.14	3.66	0.69	0.19	0.12
Threonine	50.61	93.59	133.52	245.22	256.03	42.70	93.98	157.41	235.81	258.58	19.75	< 0.001^c^	0.87	0.89
Tryptophan	46.20	41.19	43.72	39.76	39.57	44.84	41.96	40.98	41.36	42.52	2.76	0.21	0.87	0.72
Valine	242.59	238.27	261.83	249.92	239.29	250.49	226.3	239.95	247.28	253.52	10.37	0.34	0.63	0.30
*Non-essential amino acids, μmol/L*														
Alanine	437.05	468.17	390.13	446.80	490.21	423.24	413.6	404.66	451.39	419.79	28.47	0.15	0.14	0.37
Asparagine	39.87	40.07	42.51	42.70	41.25	40.86	40.77	42.00	40.47	41.52	3.75	0.94	0.93	0.98
Aspartic acid	11.57	13.91	13.14	15.50	14.86	12.77	13.22	14.47	12.80	13.77	1.30	0.37	0.59	0.37
Cysteine	193.73	189.57	204.18	200.91	211.29	195.79	200.8	207.32	195.66	201.82	7.36	0.17	0.93	0.54
Glutamate	163.27	217.79	207.69	235.01	238.14	207.22	200.5	214.63	203.51	225.17	20.14	0.12	0.85	0.26
Glutamine	452.58	483.11	490.92	491.77	485.81	438.95	478.8	500.46	533.40	477.22	31.74	0.06^‡^	0.74	0.76
Glycine	967.1	1116.3	990.8	1028.5	1108.8	939.9	914.6	1037.9	1060.6	1112.0	16.78	0.07^c^	0.40	0.18
Homocysteine	19.72	20.42	22.07	22.29	25.53	18.15	20.44	24.24	22.15	21.70	2.24	0.08^c^	0.58	0.58
Proline	185.7	194.93	183.91	206.57	197.87	186.82	180.70	188.69	198.42	187.42	10.56	0.09^c^	0.22	0.60
Serine	93.00	103.71	99.89	111.67	108.68	86.12	93.51	98.25	98.64	108.81	4.97	< 0.001^c^	0.02	0.44
Tyrosine	67.24	64.18	55.65	63.62	59.75	66.12	62.22	59.40	59.41	55.32	3.79	0.03^c^	0.45	0.74

^a^MSE, maximum standard error

^b^Thr, level of threonine; FS, feeding system; L × Thr, interaction between level of threonine and feeding system; ^c^linear effect for Thr; ^d^quadratic effect for Thr; ^e^cubic effect for Thr

### Free AAs in plasma

The dietary essential AAs (EAAs) His, Lys, and Thr (Table 5) were affected in a cubic, quadratic, and linear manner, respectively, by dietary Thr level (*P* < 0.05) but were not affected by feeding system. Methionine was not affected by dietary Thr level but was 11% higher in the IPF pigs than in the GPF pigs (*P* < 0.05). The other EAAs were not affected by dietary Thr level or feeding system. The dietary non-essential AAs (NEAAs) glutamine (Glu) tended (*P* < 0.10) to increase in a quadratic manner as a function of dietary Thr level, whereas the NEAAs glycine (Gly), proline (Pro), and homocysteine tended (*P* < 0.10) to increase linearly with the increase in dietary Thr level. Serine (Ser) increased but tyrosine (Tyr) decreased linearly with the increase in dietary Thr level (*P* < 0.05). Serine was 7% higher in the IPF pigs than in the GPF pigs (*P* < 0.05). The NEAAs Glu, glutamate, Gly, homocysteine, Pro, Ser, and Tyr increased in a linear manner as dietary Thr level increased, but only Ser was affected by the feeding system, being 4% lower in the IPF pigs than in the GPF pigs.

**Table 5:** Liver amino acid concentrations of growing barrows (25 to 42 kg body weight) fed different levels of threonine (70%, 85%, 100%, 115%, and 130% of the ideal threonine:lysine ratio of 0.65) in an individual precision-feeding (IPF) system or a group phase-feeding (GPF)

Parameter	IPF					GPF						*P*-value^b^		
	70	85	100	115	130	70	85	100	115	130	MSE^a^	Thr	FS	Thr × FS
Number of observations	5	3	6	5	5	5	5	5	5	5				
*Chemical composition* ^*c*^ *, %*														
Dry matter	28.94	27.70	28.35	28.73	28.66	28.08	28.58	28.29	28.69	29.04	0.53	0.59	0.84	0.49
Crude protein	20.44	20.28	20.35	20.57	20.72	20.34	20.55	20.34	20.77	20.26	0.33	0.84	0.92	0.78
Fat	7.11	6.21	6.35	6.17	6.44	5.91	6.77	6.69	6.41	7.43	0.51	0.72	0.53	0.19
Ash	1.48	1.51	1.50	1.51	1.48	1.47	1.47	1.47	1.59	1.47	0.04	0.21	0.93	0.54
*Essential amino acids, g/100 g of crude protein*														
Arginine	6.89	7.00	6.87	6.88	6.89	6.69	6.91	7.09	6.85	6.82	0.18	0.76	0.76	0.76
Histidine	3.01	2.91	2.92	3.03	3.04	3.03	2.92	2.99	2.93	2.88	0.08	0.69	0.49	0.48
Isoleucine	4.47	4.36	4.36	4.39	4.45	4.28	4.37	4.30	4.44	4.42	0.06	0.40	0.23	0.32
Leucine	9.00	8.76	8.83	9.01	9.00	8.86	8.82	8.86	8.87	8.82	0.11	0.50	0.23	0.62
Lysine	7.52	7.34	7.29	7.50	7.32	7.39	7.30	7.25	7.32	7.28	0.12	0.38	0.22	0.95
Methionine	3.19	3.00	2.80	3.14	3.21	2.79	2.68	2.59	3.16	2.51	0.44	0.82	0.21	0.93
Phenylalanine	5.00	4.87	4.92	5.03	5.10	4.97	4.92	4.99	4.96	4.93	0.06	0.24	0.33	0.14
Threonine	4.48	4.40	4.38	4.49	4.44	4.34	4.38	4.38	4.41	4.42	0.05	0.60	0.09	0.65
Valine	5.83	5.68	5.73	5.80	5.83	5.79	5.72	5.74	5.76	5.71	0.08	0.61	0.49	0.86
*Non-essential amino acids, g/100 g of crude protein*														
Alanine	5.76	5.64	5.69	5.73	5.71	5.71	5.62	5.68	5.69	5.61	0.06	0.39	0.22	0.94
Asparagine	10.51	10.18	10.31	10.52	10.40	10.37	10.19	10.20	10.35	10.16	0.17	0.41	0.18	0.95
Cysteine	1.14	1.24	1.33	1.23	1.19	1.15	1.18	1.24	1.23	1.25	0.06	0.21	0.65	0.69
Glutamate	12.56	11.80	11.02	11.68	12.03	12.05	11.94	11.05	11.21	11.22	0.63	0.23	0.37	0.91
Glycine	5.92	5.68	5.87	5.75	5.78	5.75	5.71	5.83	5.84	5.70	0.08	0.21	0.50	0.44
Proline	4.79	4.64	4.77	4.76	4.76	4.67	4.68	4.73	4.78	4.66	0.05	0.24	0.22	0.40
Serine	4.53	4.52	4.49	4.57	4.49	4.39	4.44	4.44	4.48	4.43	0.06	0.74	0.02	0.92
Tyrosine	4.21	4.12	4.11	4.18	4.19	4.06	4.15	4.08	4.15	4.13	0.05	0.47	0.10	0.36

^a^MSE, maximum standard error; ^*c*^*Fresh basis*

^b^Thr, level of threonine; FS, feeding system; Thr × FS, interaction between level of threonine and feeding system

### Liver AAs and chemical composition

In this growth trial (Table 6), Thr (tendency; *P* < 0.10) and Ser (*P* < 0.05) concentrations (g AA/100 g CP) in the liver were 1 and 2% higher, respectively, in the IPF pigs than in the GPF pigs. The other EAAs and NEAAs, DM, CP, fat, and ash were not affected by Thr level or feeding system or their interaction during the growing phase.

**Table 6:** Intestine amino acid concentrations of growing barrows (25 to 42 kg body weight) fed different levels of threonine (70%, 85%, 100%, 115%, and 130% of the ideal threonine:lysine ratio of 0.65) in an individual precision-feeding (IPF) system or a group phase-feeding (GPF) system

Parameter	IPF					GPF						*P*-value^2^		
	70	85	100	115	130	70	85	100	115	130	MSE^1^	Thr	FS	Thr × FS
Number of observations	5	3	6	5	5	5	5	5	5	5				
*Chemical composition* ^*^ *, %*														
Dry matter	17.14	16.82	17.26	17.09	17.44	17.04	17.39	17.20	16.76	17.25	0.33	0.67	0.90	0.63
Crude protein	12.91	12.93	13.23	13.31	13.34	13.09	13.44	13.24	12.95	13.13	0.20	0.63	0.80	0.16
Fat	3.04	2.69	2.78	2.42	2.69	2.60	2.62	2.62	2.54	2.86	0.24	0.54	0.57	0.62
Ash	0.96	0.90	0.98	1.00	0.96	0.96	1.00	1.00	0.96	0.97	0.02	0.50	0.20	0.10
*Essential amino acids, g/100 g of crude protein*														
Arginine	8.17	8.13	8.01	8.16	8.02	7.96	8.29	8.11	8.00	8.09	0.10	0.40	0.94	0.14
Histidine	2.64	2.60	2.65	2.63	2.60	2.57	2.62	2.66	2.61	2.65	0.03	0.51	0.99	0.42
Isoleucine	4.19	4.13	4.18	4.14	4.11	4.04	4.21	4.20	4.17	4.17	0.05	0.47	0.81	0.10
Leucine	8.13	8.00	8.18	8.14	8.12	7.93	8.22	8.22	8.14	8.11	0.08	0.20	0.84	0.11
Lysine	7.71	7.55	7.73	7.63	7.64	7.47	7.67	7.74	7.68	7.71	0.09	0.37	1.00	0.21
Methionine	1.80	1.81	1.57	1.81	1.57	2.17	1.88	2.01	1.85	1.62	0.21	0.35	0.09	0.68
Phenylalanine	4.46	4.39	4.51	4.50	4.49	4.38	4.48	4.50	4.45	4.49	0.04	0.12	0.73	0.19
Threonine	4.59	4.60	4.62	4.65	4.64	4.51	4.69	4.69	4.61	4.60	0.05	0.14	0.98	0.21
Valine	5.19	5.14	5.21	5.16	5.16	5.03	5.23	5.21	5.18	5.19	0.06	0.37	0.88	0.19
*Non-essential amino acids, g/100 g of crude protein*														
Alanine	6.16	6.19	6.13	6.16	6.19	6.09	6.22	6.16	6.13	6.09	0.07	0.74	0.46	0.76
Asparagine	10.92	10.87	10.97	11.04	10.83	10.63	11.00	10.96	10.72	10.92	0.11	0.31	0.21	0.06^a^
Cysteine	1.15	1.19	1.15	1.26	1.19	1.20	1.21	1.24	1.15	1.12	0.06	0.86	0.90	0.30
Glutamate	14.97	15.19	14.97	15.22	14.95	14.89	15.44	15.20	15.03	14.90	0.25	0.46	0.84	0.83
Glycine	7.96	8.23	7.79	8.04	8.08	8.00	7.99	7.90	7.90	7.83	0.19	0.65	0.38	0.75
Proline	5.74	5.84	5.71	5.80	5.84	5.71	5.82	5.75	5.72	5.71	0.09	0.65	0.37	0.83
Serine	4.79	4.78	4.82	4.85	4.82	4.74	4.90	4.90	4.73	4.79	0.05	0.26	0.96	0.08^a^
Tyrosine	4.12	4.08	4.12	4.12	4.12	3.99	4.15	4.15	4.13	4.12	0.04	0.33	0.98	0.15

^1^MSE, maximum standard error; ^*^Fresh basis

^2^Thr, level of threonine; FS, feeding system; Thr × FS, interaction between level of threonine and feeding system; ^a^cubic effect within GPF

### Intestine AAs and chemical composition

Asparagine (Asp) and Ser showed a feeding system × Thr level interaction with no effect on intestine AA composition in the IPF pigs and a cubic effect tendency (*P* < 0.10) in the GPF pigs (Table 7). Methionine tended (*P* < 0.10) to be 10% lower in the small intestinal tissue in the IPF pigs in comparison with the GPF pigs. The other EAAs and NEAAs, DM, CP, fat, and ash were not affected by Thr level or feeding system or their interaction during the growing phase. Longissimus dorsi AAs and chemical composition.

**Table 7:** Longissimus dorsi amino acid concentrations of growing barrows (25 to 42 kg body weight) fed different levels of threonine (70%, 85%, 100%, 115%, and 130% of the ideal threonine:lysine ratio of 0.65) in an individual precision-feeding (IPF) system or a group phase-feeding (GPF) system

Parameter	IPF					GPF						*P*-value^2^		
	70	85	100	115	130	70	85	100	115	130	MSE^1^	Thr	FS	Thr × FS
Number of observations	5	3	6	5	5	5	5	5	5	5				
*Chemical composition* ^*^ *, %*														
Dry matter	24.38	24.49	24.15	24.05	24.51	23.95	23.99	24.55	24.53	24.30	0.43	0.96	0.82	0.47
Crude protein	20.63	21.07	21.13	20.92	21.59	21.31	21.32	21.07	21.29	20.71	0.41	0.92	0.66	0.03^a^
Fat	2.15	2.05	1.97	1.44	1.73	1.70	1.78	1.71	1.79	1.95	0.27	0.66	0.56	0.30
Ash	1.14	1.19	1.18	1.19	1.18	1.15	1.18	1.17	1.18	1.17	0.04	0.73	0.68	1.00
Collagen	0.57	0.62	0.60	0.51	0.45	0.55	0.54	0.54	0.52	0.54	0.04	0.05^†^	0.64	0.09^c^
*Essential amino acids, g/100 g of crude protein*														
Arginine	7.38	7.51	7.34	7.33	7.19	7.40	7.41	7.34	7.40	7.49	0.12	0.75	0.36	0.36
Histidine	5.29	4.91	4.81	4.92	4.64	5.23	4.98	4.92	4.84	4.96	0.16	0.01^†^	0.38	0.50
Isoleucine	5.10	5.22	5.07	5.07	4.98	5.13	5.10	5.08	5.06	5.20	0.07	0.57	0.51	0.08^a,d^
Leucine	8.62	8.68	8.63	8.57	8.36	8.68	8.60	8.56	8.55	8.74	0.09	0.60	0.27	0.02^a,d^
Lysine	9.39	9.52	9.42	9.39	9.11	9.49	9.43	9.33	9.30	9.47	0.12	0.36	0.53	0.08^c^
Methionine	2.66	2.38	2.70	2.87	2.52	2.97	2.45	2.28	2.41	2.51	0.23	0.25	0.40	0.18
Phenylalanine	4.48	4.48	4.47	4.48	4.34	4.51	4.45	4.43	4.48	4.48	0.06	0.53	0.54	0.33
Threonine	4.89	4.91	4.92	4.92	4.70	4.92	4.92	4.85	4.86	4.94	0.07	0.42	0.30	0.03^c^
Valine	5.37	5.44	5.34	5.32	5.24	5.41	5.34	5.34	5.32	5.47	0.08	0.74	0.39	0.13
*Non-essential amino acids, g/100 g of crude protein*														
Alanine	6.03	6.10	6.04	6.05	5.81	6.12	6.04	6.03	6.00	6.08	0.07	0.14	0.18	0.02^c^
Asparagine	11.88	11.76	11.66	11.85	11.34	11.81	11.74	11.80	11.78	11.85	0.16	0.35	0.24	0.14
Cysteine	0.94	0.97	0.95	0.88	0.90	0.90	0.92	0.93	0.94	0.97	0.03	0.50	0.69	0.05^a,b^
Glutamate	17.42	17.72	17.73	17.81	16.28	16.98	17.78	17.52	17.45	18.01	0.56	0.55	0.58	0.09^c^
Glycine	4.75	4.76	4.70	4.76	4.57	4.79	4.73	4.80	4.75	4.81	0.07	0.68	0.08	0.18
Proline	4.00	4.02	4.02	4.03	3.90	4.06	4.03	4.05	4.00	4.08	0.06	0.86	0.10	0.20
Serine	4.19	4.13	4.22	4.21	4.04	4.23	4.18	4.16	4.15	4.17	0.05	0.13	0.48	0.16
Tyrosine	4.16	4.19	4.16	4.18	4.04	4.20	4.16	4.14	4.16	4.19	0.06	0.65	0.47	0.32

^1^MSE, maximum standard error; ^*^Fresh basis

^2^Thr, level of threonine; FS, feeding system; Thr × FS, interaction between level of threonine and feeding system; ^†^linear effect for Thr; ^a^linear effect within IPF; ^b^linear effect within GPF; ^c^quadratic effect within IPF; ^d^quadratic effect within GPF;

Histidine decreased linearly in the longissimus dorsi as dietary Thr level increased (*P* < 0.05), independent of feeding system (Table 8). Isoleucine (tendency; *P* < 0.10) and Leu decreased *P* < 0.05 linearly in the IPF pigs and in a quadratic manner in the GPF pigs. Lysine (*P* < 0.10), glutamate (*P* < 0.10), Thr (*P* < 0.05), and alanine (Ala) (*P* < 0.05) increased in a quadratic manner in the IPF pigs as dietary Thr level increased, but those AA were not affected in the GPF pigs. Cysteine tended to decrease (*P* < 0.10) linearly in the IPF pigs, whereas it tended to increase linearly in the GPF pigs. Glycine tended to be 1.4% higher (*P* < 0.10) in the GPF pigs than in the IPF pigs. Collagen in the longissimus dorsi decreased (*P* < 0.05) with the increase in dietary Thr level, independent of feeding system. The other EAAs and NEAAs, DM, CP, fat, and ash were not affected by Thr level or feeding system or their interaction during the growing phase.

**Table 8:** Carcass muscle amino acid concentrations (without longissimus dorsi) of growing barrows (25 to 42 kg body weight) fed different levels of threonine (70%, 85%, 100%, 115%, and 130% of the ideal threonine:lysine ratio of 0.65) in an individual precision-feeding (IPF) system or a group phase-feeding (GPF) system

Parameter	IPF					GPF						*P*-value^2^		
	70	85	100	115	130	70	85	100	115	130	MSE^1^	Thr	FS	Thr× FS
Number of observations	5	3	6	5	5	5	5	5	5	5				
*Chemical composition* ^*^ *, %*														
Dry matter	31.10	30.39	29.84	29.59	29.94	29.37	30.22	30.52	29.84	29.73	0.76	0.82	0.55	0.33
Crude protein	17.40	17.54	17.82	17.78	18.39	18.24	17.93	18.06	17.87	18.18	0.26	0.09^†^	0.05	0.13
Fat	12.84	12.19	11.46	10.79	11.25	10.88	11.67	10.72	11.05	11.10	1.02	0.64	0.23	0.70
Ash	0.99	1.00	1.02	1.01	1.00	1.01	0.99	1.00	1.00	0.99	0.02	0.91	0.48	0.86
Collagen	1.61	1.60	1.60	1.66	1.63	1.56	1.66	1.73	1.61	1.61	0.08	0.76	0.69	0.41
*Essential amino acids, g/100 g of crude protein*														
Arginine	7.40	6.63	7.12	7.62	6.99	7.13	7.93	7.63	7.11	7.11	0.29	0.63	0.12	0.01^a,b^
Histidine	4.45	3.68	3.94	4.30	4.09	4.27	4.54	4.28	3.88	3.99	0.23	0.48	0.39	0.02^a,c^
Isoleucine	4.80	4.39	4.74	4.88	4.48	4.72	5.19	4.99	4.68	4.70	0.19	0.48	0.05	0.03^a,b^
Leucine	8.26	7.57	8.10	8.46	7.79	8.12	9.03	8.22	8.10	8.10	0.26	0.61	0.07	0.01^a,b^
Lysine	8.74	8.02	8.58	9.05	8.29	8.64	9.51	9.18	8.59	8.63	0.38	0.67	0.06	0.04^a,c^
Methionine	2.56	2.19	2.28	2.87	2.73	2.24	2.87	2.36	2.66	2.60	0.26	0.15	0.87	0.19
Phenylalanine	4.37	3.95	4.23	4.44	4.10	4.29	4.73	4.53	4.23	4.25	0.18	0.66	0.04	0.02^a,b^
Threonine	4.56	4.20	4.54	4.76	4.38	4.57	5.09	4.56	4.53	4.51	0.17	0.61	0.07	0.01^a,b^
Valine	5.21	4.73	5.09	5.30	4.84	5.12	5.62	5.39	5.09	5.06	0.17	0.44	0.04	0.03^a,b^
*Non-essential amino acids, g/100 g of crude protein*														
Alanine	6.32	5.74	6.12	6.44	5.91	6.22	6.78	6.61	6.19	6.06	0.26	0.43	0.05	0.04^a,d^
Asparagine	11.27	10.15	10.92	11.30	10.46	10.98	12.08	11.58	10.85	10.81	0.47	0.54	0.07	0.03^a,c^
Cysteine	0.95	0.88	0.93	0.95	0.87	0.96	1.04	1.03	0.91	0.92	0.05	0.33	0.04	0.20
Glutamate	15.37	13.44	15.39	13.52	13.37	14.96	15.28	15.23	14.10	13.60	1.20	0.21	0.49	0.82
Glycine	5.97	5.48	5.80	6.14	5.75	5.97	6.32	6.47	5.98	5.74	0.27	0.44	0.06	0.11
Proline	4.67	4.29	4.56	4.82	4.50	4.66	5.08	5.01	4.65	4.56	0.20	0.55	0.03	0.04^a,d^
Serine	4.11	3.74	4.02	4.21	3.92	4.03	4.50	4.31	4.05	3.97	0.18	0.61	0.07	0.03^a,b^
Tyrosine	4.00	3.63	3.92	4.04	3.74	3.91	4.35	4.12	3.85	3.89	0.16	0.55	0.06	0.02^a,b^

^1^MSE, maximum standard error; ^*^ *Fresh basis*

^2^Thr, level of threonine; FS, feeding system; Thr × FS, interaction between level of threonine and feeding system; ^†^linear effect for Thr; ^a^cubic effect within IPF; ^b^cubic effect within GPF; ^c^tendency for a cubic effect within GPF; ^d^quadratic effect within GPF

### Pool of carcass muscle AAs and chemical composition

In the pool from the right half of the carcass, the EAAs Arg, Iso, Leu, phenylalanine, Thr, and Val and the NEAAs Ser and Tyr showed an interaction between dietary Thr level and feeding system (*P* < 0.05), decreasing in a cubic manner in the IPF pigs and increasing in a cubic manner in the GPF pigs (Table 9). The EAAs His and Lys and the NEAA Asp also showed an interaction between dietary Thr level and feeding system (*P* < 0.05), with a cubic decrease in concentration in the IPF pigs and a tendency (*P* < 0.10) toward a cubic increase in the GPF pigs. The NEAAs Ala and Pro were affected by an interaction between dietary Thr level and feeding system (*P* < 0.05), with the concentration decreasing in a cubic manner in the IPF pigs and increasing in a quadratic manner in the GPF pigs. Proline (*P* < 0.05), phenylalanine and Val (*P* < 0.05) and Leu (*P* < 0.10), were 5%, 4%, 3%, respectively, higher in the GPF pigs than the IPF pigs. Threonine, Lys, Iso, Ala, Asp, Ser and Tyr were 4% (*P* < 0.10) higher in the GPF pigs than the IPF pigs. Cysteine (*P* < 0.05) and Gly (*P* < 0.10) were 6% and 4% higher, respectively, in the GPF pigs than the IPF pigs, and these AAs were not affected by dietary Thr level. Glutamate, DM, ash, fat, and collagen were not affected by Thr level or feeding system or their interaction during the growing phase. However, CP tended (*P* < 0.10) to be 1.5% higher in the GPF pigs than in the IPF pigs.

**Table 9:** Blood plasma biochemical parameters of growing barrows (25 to 42 kg body weight) fed different levels of threonine (70%, 85%, 100%, 115%, and 130% of the ideal threonine:lysine ratio of 0.65) in an individual precision-feeding (IPF) system or a group phase-feeding (GPF) system

Parameter	IPF					GPF						*P*-value^2^		
	70	85	100	115	130	70	85	100	115	130	MSE^1^	L	FS	L × FS
Number of observations	10	8	11	10	10	11	10	11	11	11				
Urea, μmol/L	2.70	1.98	2.38	2.19	2.77	2.74	2.04	2.34	2.07	2.40	0.23	< 0.01^‡^	0.51	0.83
Albumin, g/L	27.80	26.56	32.12	31.59	33.51	29.50	31.44	32.25	31.63	31.19	1.25	< 0.01^†^	0.19	0.03
Creatinine, μmol/L	116.85	114.69	112.68	110.85	117.50	117.50	116.91	115.35	112.41	119.41	3.69	0.25	0.39	1.00
Lactic acid dehydrogenase, U/L	585.34	581.88	535.13	532.60	570.35	524.96	485.73	537.90	468.60	557.14	47.66	0.53	0.06	0.72
Total protein, g/L	62.65	64.26	65.33	66.90	67.48	61.86	65.56	64.37	66.00	66.13	1.52	0.01^†^	0.52	0.89
Aspartate aminotransferase, U/L	36.75	44.96	38.35	43.80	43.09	36.89	37.50	36.60	36.48	44.21	3.50	0.08^†^	0.08	0.34
Alanine aminotransferase, U/L	47.50	40.79	39.73	40.00	38.39	41.14	45.05	44.37	36.06	43.90	3.03	0.14	0.60	0.04^a,b^
Creatine kinase, U/L	1083	1561	1227	1822	1918	1108	1244	1562	1015	2172	412	0.15	0.67	0.52
Immunoglobulin G, μg/mL	11.29	11.28	9.93	11.90	10.98	9.71	10.90	9.48	11.31	11.36	1.18	0.19	0.33	0.84
C-reactive protein, μg/mL	9.25	13.02	9.98	18.35	24.78	13.88	15.81	18.46	22.82	12.68	3.56	0.05^†^	0.26	0.01^a,c^

^1^MSE, maximum standard error

^2^L, level of threonine; FS, feeding system; L × FS, interaction between level of threonine and feeding system; ^†^linear effect for L; ^‡^quadratic effect for L; ^a^linear effect within IPF; ^b^cubic effect within GPF; ^c^quadratic effect within GPF

## Discussion

### Performance is affected by Thr level

Threonine levels did not affect ADFI during this growing phase, a result that is consistent with the literature [28, 31, 32]. The improved G:F ratio is due to the linear increase in ADG without changes in the ADFI. Normally, pigs fed in conventional group-feeding systems receive on average during the overall growing and finishing period 26% more Lys than pigs fed daily tailored diets do [7]. However, SID Lys intake was similar in this trial between the GPF and the IPF pigs. This similarity was due to the fact that dietary SID Lys concentration was decreased by 10% in the GPF pigs to ensure that Lys was the second-limiting AA, whereas each day, the IPF pigs received the estimated amount of SID Lys required for maintenance and growth. As well, SID Lys requirement for GPF was precisely adjusted knowing individual requirements, making this concentration (SID Lys 0.88%), similar to the average SID Lys provided to IPF pigs (SID Lys of 0.85%). It was this artefact that allowed us to compare both programs in equal basis avoiding Lys to drive the protein response. Still, SID Thr intake increased linearly, as expected, due to the increase in Thr concentration in the feeds.

During this growth trial, the linear increase in dietary Thr concentration allowed PD to increase linearly in both feeding systems, in line with the literature [28]. However, PD was not affected by feeding system, whereas compared with the 100% level of SID Thr intake, 30% Thr restriction resulted in only 12% decrease of PD. Previously, Andretta et al. [7] showed that moving from conventional to precision feeding systems does not affect growing pigs PD or performance. The percentage of protein or lipids in daily gain during the growing phase was not affected by dietary treatments even at the lower levels of PD. Cloutier et al. [7] observed a tendency of decrease in the percentage of protein in daily gain but no effect in LipD in the pigs receiving a diet 30% deficient in SID Lys. A higher backfat thickness and lower lean percentage resulted from feeding pigs with Lys deficient diets [33]. It is however expected that when dietary energy levels are sufficient to promote maximum PD, but that an essential AA is limiting, PD would be reduced and the energy that is not used for protein synthesis would be stored in the form of lipids [34]. Still that growing pigs have high PD potential, but also that there is a great variation between animals. This large variation with respect to the percentage of protein in daily gain may have prevented the increase in LipD that is expected when PD is limited with a similar energy intake.

Estimated Thr and Lys efficiencies of utilization increased to nearly 100% at lower AA intake levels, with the most efficient animals in terms of AA utilization generating values over 100% of AA retention. Threonine efficiency values of 91% [35] and 86% [28] and Lys efficiency values of 107% and 101% [36] are found in the literature when pigs are fed AA-deficient diets. Lysine efficiency seems to increase with the level of dietary Lys deficiency, indicating that pigs are more efficient in utilizing Lys when they are fed below requirements [37]. The Lys and Thr efficiencies values found in this study are higher than those found in the literature, which are around 72% for Lys and 62% for Thr [29]. The difference between the values observed in this trial and those in the literature may be the result of metabolic or experimental factors [38]. Thus, the increase in Lys and Thr efficiency values when pigs are fed Lys- and Thr-deficient diets may result in part from the difficulties of estimating maintenance requirements [28], which may be different from one animal to another because of each individual animal’s metabolism. Furthermore, a constant efficiency value is generally proposed because body protein AA concentration is assumed to be constant and independent of the pig’s age, nutrient intake, and lean and fat growth rates [28]. Therefore, high AA efficiency of utilization might result from the fact that these efficiencies values were obtained through a back calculation using the observed PD in the pigs but assuming the Lys concentration constant as 6.9% of the protein. This constant AA concentration in protein seems to be an invalid assumption, given that protein and energy levels [39], age [11], sulfur AA deficiency [12, 40], Thr deficiency [13] or excess, and genetics [41] can change body AA composition. The most metabolically efficient pigs may use several mechanisms, such as decreased protein degradation, increased AA absorption in the small intestinal tissue, and increased absorption of AAs from plasma proteins, to cope with lower AA intake, thereby contributing to the higher AA efficiency.

### Amino acid ratios cannot be used for precision feeding

In this study, the estimated ideal Thr:Lys ratio was 65% for the GPF system, but the ideal ratio for pigs fed daily tailored diets was not clear, due the linear response to increasing Thr:Lys. Ratios based on the ideal protein profile have been assumed to be a practical way to formulate diets for non-ruminants, decreasing the use of CP [24, 42, 43]. There was concern, however, about whether these constant AA ratios could also be applied for IPF. In this feeding system, the required concentration of SID Lys is estimated individually for each pig using individual DFI and BW information. The other EAAs and the pool of NEAAs are supplied in this method using conventional ideal AA ratios. The proportional decrease in Thr as Lys requirement decreased seemed to limit the performance of the IPF system when a Thr:Lys ratio of 65% was used. Our findings point to the conclusion that for IPF, independent estimates of Thr and possibly other AAs requirements, are required.

Establishing recommendations for AA requirements can be hampered by the differences between individuals and the availability of dietary nutrients. More important than determining an acceptable ratio between AAs is understanding the factors that are at the origin of the differences between animals. In this trial, we observed a large variation within treatments in both feeding systems. This within-treatment variation might be associated with between-animal variation, as well as with experimental and metabolic factors. In situations where the AA intake is not sufficient to support maximum growth, the growth rate is reduced and the AA composition of muscles is changed [11]. It is possible in such situations that the AA metabolism is affected and that this effect is modulated by the composition and amount of AAs supplied in the diet. In other words, the animal does not have a requirement but rather a response to AA intake, thereby generating variance.

### Metabolism is affected by feeding system and Thr levels

Normally, AST, ALT, CK, and creatinine are the recommended variables used for identifying liver and kidney damage or failure. In this study, these biochemical variables were within the expected ranges for growing pigs [44], and therefore, the plasma enzymatic changes in AST, ALT, and CK observed in this trial are associated more likely with changes in total muscle tissue mass and metabolism than with liver damage. The AST in plasma was 8% higher in the IPF pigs than in the GPF pigs, pointing to possible muscle breakdown. With the lowest levels of Thr intake in the IPF system (i.e., 30% below the requirement), ALT activity and urea in plasma were increased, suggesting an increase in the deamination of Ala and other AAs and in urea synthesis. Meanwhile, in the GPF system, ALT in plasma increased in a cubic manner and urea decreased in a quadratic manner with the increase in dietary Thr level. Thus, increased ALT with linear plasma urea increase within IPF at lower levels of dietary Thr can indicate that pigs restrictive treatments had lower protein synthesis or higher AA catabolism.

C-reactive protein was within normal values for healthy pigs [44]. Nonetheless, Thr in plasma increased with the increase in Thr intake, reflecting a linear increase in CRP in the IPF pigs and a quadratic increase in CRP in the GPF pigs. C-reactive protein is a major acute-phase protein in pigs exposed to health challenges [45]. But more importantly, this protein is composed mainly of Ser (9.62%), Gly (7.48%) and Thr (6.4%) [46]. Because Thr and its products are important components of CRP, it is possible that more CRP was synthesized at higher levels of Thr intake and that, at lower levels of Thr intake, CRP was degraded to provide Thr, serine, and Gly for protein synthesis. It is therefore likely that the increases in plasma Ser, Gly, and Thr favoured the synthesis of CRP. The low levels of albumin in plasma observed in the pigs in the Thr-deprived dietary treatments may point to albumin synthesis reduction. The rate of albumin synthesis is reduced in cases of malnutrition, malabsorption, or maldigestion [47], what could result from Thr deficient diets. Plasma albumin accounts for 0.5% of total body proteins, as it is the major blood protein and an important protein carrier in plasma [48]. The decrease in albumin concentration in plasma could have contributed to the reduction of the supply of AAs for the natural turnover of protein in peripheral tissues [45]. In general, we observed a linear increase in plasma proteins (albumin, total protein, and CRP) with the plasmatic increase of Thr. Albumin prevents irreversible oxidative losses by capturing excess AAs and transporting them to peripheral tissues, in order to sustain local protein synthesis [49]. When the concentration of AAs in tissue cells decreases, plasma proteins are transported into tissue cells to provide AAs and ensure cellular equilibrium [50]. Therefore, when Thr deficient diets are provided to pigs, low plasma protein concentration may occurs due use of these proteins to maintain to peripheral tissues protein synthesis; still, Thr deficiency might decrease the rate of plasma protein synthesis. Both mechanisms could be used by the metabolism to increase the efficiency with which it uses the limiting AA, as has been observed in this and other trial [52] where pigs were fed at lower levels of Thr.

Higher concentrations of plasma Lys and His were found in the pigs fed at low levels of dietary Thr in both feeding systems. When one AA is limiting in the diet (Thr in our case), some essential AAs such as Lys [13] and His [11] will increase in plasma, probably due to their low utilization for net PD [52]. The linear increase in the plasma concentrations of Gly and serine in both feeding systems, might be due to the Thr linear increase in plasma. Threonine in pigs is oxidized in the liver and pancreas into Gly and Ser [53]. Plasma Met and Ser levels were 11% and 7% higher, respectively, in the IPF pigs than in the GPF pigs. This difference might suggest higher oxidation of Gly in Ser in IPF system even if the rate of conversion of Gly to Ser seems limited by intestinal capacity in young pigs [54] or higher oxidation of Glu in Ser. The higher plasma Met is likely due to lower Met retention in the small intestinal tissue of the IPF pigs, which was 10% lower than in the GPF pigs.

### Splanchnic tissue tends to be preserved over AA restriction

Amino acid concentration and protein content in the small intestinal tissue and liver were not affected by dietary Thr levels, with the exception of Ser and a trend for Thr in the liver, which were 2% and 1% higher, respectively, in the IPF pigs than in the GPF pigs. Other studies in which animals were fed in conventional group-feeding systems with diets deficient in either Thr [13] or sulfur AAs [11, 12] showed low or no impact on AA concentration in the small intestinal tissue. This lack of effect of dietary AA deficiency on small intestinal tissue AA concentration can be attributed to the fact that most of the AAs retained in the proximal part of the small intestine come from the diet [55] and that absorbed dietary AAs are used first by the splanchnic tissues [12]. We can speculate that splanchnic tissues are protected from AA deficiency because of the dietary AA pathway, which reaches the liver via the portal vein after crossing the intestinal walls. Indeed, the liver and intestine are the main sites for AA metabolism in mammals. The metabolism seems to protect the integrity of these organs before other tissues, because the liver and intestine receive the absorbed AAs before others such as the skeletal tissues, thus resulting in smaller variation in AA splanchnic tissue composition. Hamard et al. [13] found higher Thr retention in the liver and colon of Thr-deficient pigs. It is plausible that the IPF pigs that received decreasing concentrations of AAs throughout the growing period developed additional metabolic mechanisms to cope with Thr deficiency, such as higher Thr retention. The lower Thr concentration and the tendency toward lower Ser concentrations found in the pool of skeletal muscles of the IPF pigs may indicate that the organism tried to retain the limiting AA for protein synthesis in the liver in order to optimize protein synthesis at the moment of AA availability. The higher levels of AST in the IPF pigs in this and another study [51] may signal skeletal muscle protein breakdown for resynthesis during AA restriction, supporting the idea that pigs use diverse mechanisms to cope with AA deficiency.

### Muscle AA composition is affected differently by Thr restriction and feeding systems

In the IPF and GPF systems evaluated in this study, muscle AA concentrations were affected by Thr restriction in an opposite cubic manner. Conde-Aguilera et al. [40] found that sulfur AA restriction had little effect on carcass AA concentration when the trial duration was 10 d, but longer periods of restriction affected muscle protein content and AA concentration [11]. In a 14-day experiment, Hamard et al. [13] found no effect on protein content and little effect on AA concentration in carcasses muscles, with the exception of Thr, which decreased in animals with a 30% Thr restriction. The 21-day length of the present trial, which is 7 d longer than previous studies [13, 40], can explain the effects of Thr restriction on muscle AA concentration and protein content observed in our study. Protein concentration in the longissimus dorsi increased linearly in the IPF pigs and was not affected in the GPF pigs. In the longissimus dorsi, protein concentration was, on average, equal between the two systems, whereas protein concentration in the pool of carcass muscles tended to be 1.5% higher in the GPF pigs than in the IPF pigs. This lower protein concentration signals that the IPF pigs were more affected by Thr restriction than the GPF pigs were. Nutrient requirements in growing pigs change rapidly over the growing period, and animals fed in conventional GPF systems may have limiting supplies of AAs at the beginning of the phase but not necessarily throughout the entire period [23]. In an in silico study, Hauschild et al. [23] demonstrated that the optimal SID Lys concentration to be served in a 28-day feeding phase underfed part of the population during half of the period but overfed another part of the population. In contrast, the requirements of pigs fed daily tailored diets are adjusted every day, and AA concentration decreases over time [6, 56]. Thus, the IPF pigs that were restricted in Thr on the first day of the trial were restricted for the entire experimental period. This might explain the high impact of AA restrictions on protein and AA concentrations in the IPF pigs in comparison with the GPF pigs.

The difference in AA concentration among different tissues, mainly among different muscles, can be due to growth hormone action; in other words, a nutritional restriction can downregulate growth hormone mRNA receptors in the liver but also upregulate them in skeletal tissues [57]. More than feed intake and energy balance, other nutrients can regulate growth hormones. In the longissimus dorsi, for example, a Thr deficiency can upregulate growth hormone [58]. Growth hormone was not measured in this trial, but it can be speculated that the effect of Thr restriction on the AA and protein concentrations observed in this trial was also mediated by hormonal changes. Collagen has been considered a source of NEAA reserves, and in situations where less Thr is available, proteins that are poorer in this AA, such as collagen, can be synthesized. Threonine restriction did not affect collagen synthesis in the GPF pigs in this trial, a result that is in agreement with those of previous studies [11, 13] in which pigs were fed in conventional group-feeding systems. The results of the present trial seem to indicate, however, that dietary Thr can affect collagen formation in pigs in an IPF system. It is possible that the IPF pigs developed several mechanisms to cope with Thr deficiency, such as collagen synthesis along with increased AA retention in the liver, as well as the use of plasma proteins as sources of AAs for peripheral tissues during AA restriction.

## Conclusions

The growth performance of growing pigs in this trial was affected by the Thr supply but not by the feeding systems under study. Dietary Thr deficiency decreased plasma proteins whereas increased collagen in the Longissimus dorsi. In addition, Thr deficiency impaired empty body composition by changing AA concentration and decreasing carcass protein in the IPF pigs in comparison with the GPF pigs. The level of dietary Thr estimated using non-linear models to optimize PD was different between the feeding systems, with the pigs in the IPF system having Thr:Lys ratio requirements that were at least 30% higher than those of the pigs in the conventional GPF system. The results of this trial show that AA requirements vary between individual pigs and cannot be accurately estimated based on traditional AA:Lys ratio studies. Furthermore, the results of this trial indicate that pigs have great capacity to deal with excess and limited AA resources, by limiting PD and changing AA composition differently among body tissues. Under limiting AA conditions, pigs modulate to some extent the utilization and retention of the limiting resource in order to maintain its natural functions in a normal manner.

## Abbreviations

AA: Amino Acid
AAs: Amino Acids
ADFI: Average Daily Feed Intake
ADG: Average Daily Gain
AIPF: Automatic and Intelligent Precision Feeding®
Ala: Alinine
ALT: Alanine Aminotransferase
Asp: Aspargine
AST: Aspartate Aminotransferase
BW: Body Weight
CP: Crude Protein
CRP: C-Reactive Protein
DFI: Individual Daily Feed intake
DM: Dry Matter
DXA: Dual-Energy X-Ray Absorptiometry
EAA: Essential Amino Acids
FS: Feeding System
g: Grams
g/d: Grams Per Day
G:F: Gain: Feed Ratio
Gly: Glycine
GPF: Group Phase Feeding
GPF100: Group phase-feeding with 100% of threonine
GPF115: Group phase-feeding with 115% of threonine
GPF130: Group phase-feeding with 130% of threonine
GPF70: Group phase-feeding with 70% of threonine
GPF85: Group phase-feeding with 85% of threonine
His: Histidine
IPF: Individual Precision Feeding
IPF100: Individual Precision Feeding with 100%of Threonine
IPF115: Individual Precision Feeding with 115% of Threonine
IPF130: Individual Precision Feeding with 130% of Threonine
IPF70: Individual Precision Feeding with 70% of Threonine
IPF85: Individual Precision Feeding with 85% of Threonine
Iso: Isoleucine
kg: Kilograms
L × F: Interaction Level and Feeding System
L: Level
Leu: Leucine
LipD: Lipid Deposition
Lys: Lysine
Met: Methionine
MSE: Maximum Standard Error
N: Nitrogen
NE: Net Energy
NEAA: Non-Essential Amino Acids
PD: Protein Deposition
R.S.E.: Residual Standard Error
See: Standard Error of the Estimation
Ser: Serine
SID: Standardized Ileal Digestible
Thr: Threonine
